# Soil microbiome analysis of a northeastern deciduous forest in SUNY Old Westbury, Long Island, New York

**DOI:** 10.17912/micropub.biology.001884

**Published:** 2026-01-16

**Authors:** Fernando Emilio Nieto Fernandez, Patricia Roccanova, Bettina Fantal-Pinckombe, Raymond Catapano

**Affiliations:** 1 Biological Sciences, SUNY Old Westbury; 2 Science and Technology Entry Program, SUNY Old Westbury; 3 Science Department, Westbury High School

## Abstract

We studied spatial changes in soil bacterial microbiome composition and diversity in a 111 acres old growth mixed hardwood forest plot in Long Island, NY. Forty soil samples were collected from four forest transects across the forest plot representing various soil features, and dominant vegetation. Three phyla account for 91% of the bacteria in the samples, Acidobacteriota (43%), Proteobacteriota (30%), and Actinobacteriota (18%). We also found 16 different classes and 33 orders. Sites dominated by black birch, *Betula lenta* were significant more diverse than all other sites. We also found significant differences in microbiome composition based on pH and vegetation.

**Figure 1. Analysis of the bacterial microbiome diversity across vegetational and soil gradients in an northeastern deciduous forest at SUNY Old Westbury:**
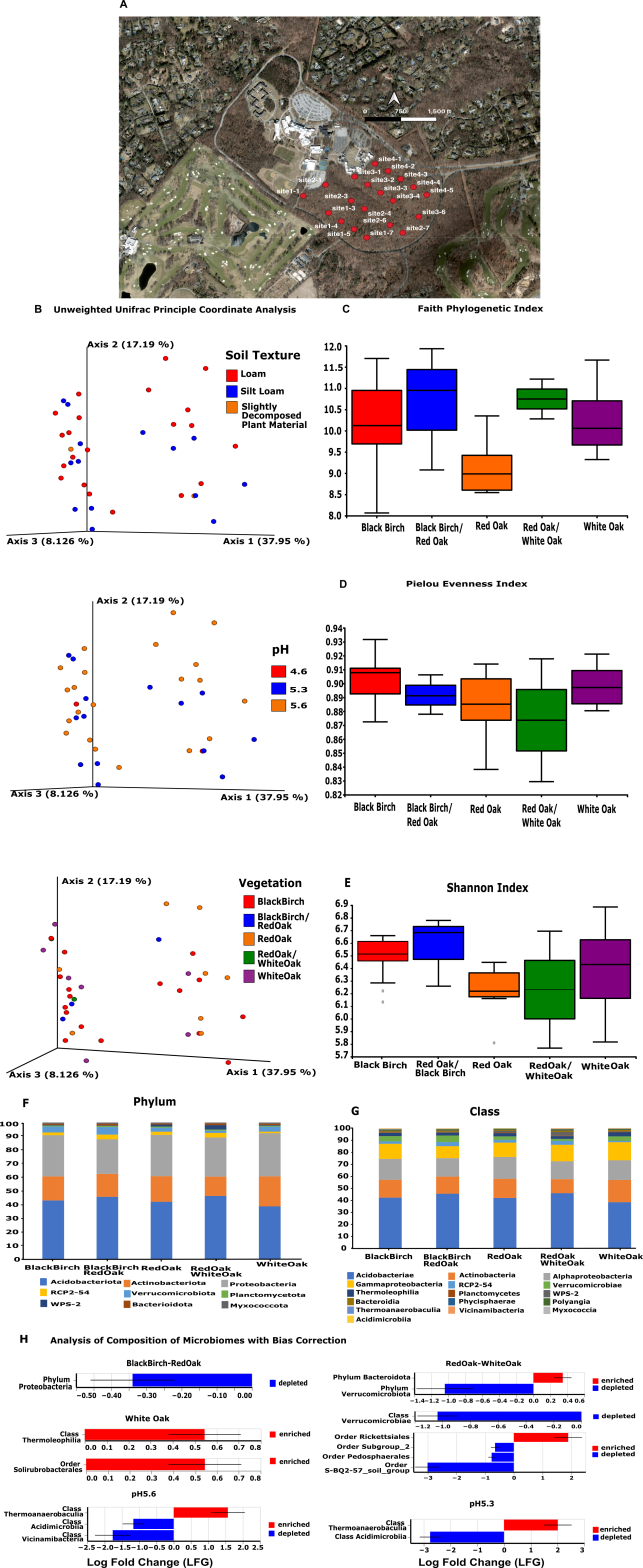
A) A map of the study site showing 112 acres of northeastern deciduous forest located on the SUNY Old Westbury campus. Samples were taken from five sites located four in each of four north to south transects. B) Beta-diversity unweighted unifrac principal coordinate analysis plot showing samples labeled based on dominant vegetation, pH and soil texture . The samples were grouped in two separate clusters but none of the variables examined, including vegetation, pH and soil texture were able to explain this clustering pattern. C) Box and whiskers plot for the Faith Phylogenetic alpha diversity index. Based on the Kruskal Wallis Test there are no significant differences in phylogenetic based diversity among samples from locations with different dominant tree canopy. D) Box and whiskers plot for the Pielou Evenness alpha diversity index. Based on the Kruskal Wallis Test there are no significant differences in evenness among samples from locations with different dominant tree canopy. E) Box and whiskers plot for the Shannon alpha diversity index. Based on the Kruskal Wallis Test there are significant differences in diversity among samples from locations with black birch and red oak as the dominant tree canopy. F) Taxonomic bar plot showing the relative percentage of the top nine phyla based on dominant tree canopy. Nine different phyla are present, three of them account for 91% of the bacteria in the samples, Acidobacteriota (43%), Proteobacteriota (30%), and Actinobacteriota (18%). The other less abundant phyla are Verrucomicrobiota (4%), RCP2-54 (2%), Bacterioidota (0.95%), Myxococcota (0.83%), WPS-2 (0.79%), and Plantomycetota (0.73%). G) Taxonomic bar plot showing the relative percentage of the top eighteen classes based on dominant tree canopy. We found eighteen different classes with at least 1% relative abundance, Acidobacteriae (42%), Actinobacteria (16%), Alphaproteobacteria (17%), Gammaproteobacteria (13%), RCP2-54 (2.10%), Verrucomicrobiae (3.92%), and Thermoleophilia (2.75%) H) Analysis of Composition of the Microbiome with Bias Correction (ANCOM-BC) revealed significant differences in relative abundance for several taxa among sites with different vegetational composition and pH. Phylum *Proteobacteria* was significantly depleted in sites dominated by a combination of black birch and red oak (p=0.05; figure 1H). Sites dominated by red oak and white oak showed a significant depletion of the phylum *Verrucomicrobiota,* class *Verrucomicrobiae,* and *orders Pedosphaerales* and *S-BQ2-57-soil-group,* and *an enrichment of the* phylum *Bacteroidota* and the order *Rickettsiales.* On sites dominated by white oak the class *Thermoleophilia* and the order *Solirubrobacteriales* were significantly enriched (p=0.05). In soils with pH 5.6, class *Thermoanaerobaculia* is enriched and classes *Acidomicrobiia* and *Vicinamibacteria* are depleted (p=0.05). Soils with pH 5.3 also show enrichment of class *Thermoanaerobaculia* and depletion of class *Acidomicrobiia* (p=0.05) *.*

## Description

Forests cover 30% of the Earth's land surface, accounting for 50% of the primary production and 45% of the carbon on land (Keenan et al., 2015, Llado et al., 2018, Suzuki et al. 2004). They provide a stratified habitat for many plants and animals, and many services of economic value to humans like timber and recreational facilities (Cardenas et al., 2015, Wood et al., 2017). Deciduous forests account for 5.3 % of earth’s land surface and their net primary productivity ranges from 600–1500 g m ^-2^ yr ^-1^ (Vasseur, 2012; Forseth, 2010). Historically deciduous forests have been exploited for logging, clearing for agricultural uses and eventually urbanization (Vasseur, 2012). The soil microbiome plays a major role in biogeochemical nutrient cycling facilitating the movement of essential macronutrients between different pools like the atmosphere, plants and the soil through enzymatic processes like nitrification, nitrogen fixation, decomposition and mineralization (Uroz et al., 2016; Lladó et al., 2017).

Soil microbial communities are very diverse and variable spatially and temporally (Llado et al., 2017, Llado et al. 2028, Peng et al. 2022, Uroz et al. 2016). The turnover rate of fungal communities is greater than that of bacteria in soils from mixed forests and it is mostly driven by species replacement rather than taxa gains or losses (Martinovic et al, 2021). The temporal changes also differ among fungal and bacterial guilds. They are also affected by soil characteristics like pH, texture, organic matter content and water capacity and biotic factors like plant roots and associated mycorrhizal activity, aboveground litter, and other decomposing organic matter (Thoms et al, 2010, Llado et al. 2018). Soil characteristics (particularly pH) have been frequently reported as strong determinants of microbial diversity (Lauber et al., 2008; Kaiser et al., 2016). Tree species have also been shown to exhibit in some instances a stronger impact on community structure (Bonito et al., 2014; Dukunde et al., 2022; Galazka et al., 2022; Staszel-Szlachta et al., 2024). Prada-Salcedo et al. (2022) showed that soil depth had a greater impact on bacterial diversity whereas tree species composition had a more significant effect on fungal community diversity. Presence of leaf litter from different tree species has also been shown to affect soil bacterial diversity and activity (Pfeiffer et al., 2013). Micronutrients have also been shown to have a significant effect on microbiome composition in agricultural soils, but the effect was more significant on fungi and protists that bacteria (Peng et al., 2022). Understanding what factors impact soil microbial community structure is germane for predicting how bacteria-mediated processes influence ecosystem responses to environmental changes (Nemergut et al., 2014; Thoms et al., 2010; Lladó et al., 2018).

In our study we focused on the spatial changes in soil bacterial microbiome composition and diversity in a 112 acre old growth mixed hardwood forest plot in SUNY Old Westbury campus, Long Island, New York. We specifically looked at the effect of above ground dominant vegetation, pH, organic matter, water capacity, soil texture and sampling year on bacterial community diversity and taxa composition. Soil physicochemical parameters were entered as part of the metadata file for downstream analysis using Qiime2.

Nine different phyla are present across all samples, three of them account for 91% of the bacteria in the samples, *Acidobacteriota* (43%), *Proteobacteriota* (30%), and *Actinobacteriota* (18%). The other less abundant phyla are *Verrucomicrobiota* (4%), *RCP2-54* (2%), *Bacterioidota* (0.95%), *Myxococcota* (0.83%), *WPS-2* (0.79%), and *Plantomycetota* (0.73%) (figure 1G). We found eighteen different classes, those with at least one percent relative abundance are *Acidobacteriae* (42%), *Actinobacteria* (16%), *Alphaproteobacteria* (17%), *Gammaproteobacteria* (13%), *RCP2-54* (2.10%), *Verrucomicrobiae* (3.92%), and *Thermoleophilia* (2.75%)(figure 1F). And thirty-three different orders, those with at least one percent relative abundance, are *Acidobacteriales* (29%), *Frankiales* (11.5%), *Acidobacteriae* -Subgroup-2 (12.5%), *Corynebacteriales* (3.94%), *Rhizobiales* (9.85%), *Elsterales* (4.16%), WD260 (5.15%), RCP2-54 (2.10%), *Gammaproteobacteria-Incertae-Sedis* (5.95%), *Pedosphaerales* (2.73%), *Chthoniobacterales* (1%), *Solirubrobacterales* (2.75%), and *Acetobacterales* (1.59%). Orders with less than one percent relative abundance included WPS-2, *Burkholderiales, Rickettsiales, Chitinophagales, Tepidisphaerales, Polyangiales, JG36-TzT-191, Xanthomonadales, S-BQ2-57-soil-group, Thermoanaerobaculales, Vicinamibacterales, Caulobacteriales, Myxococcales, IMCC26256, Catenulisporales, and Planctomycetales.*

Based on the Kruskal-Wallis test, we only found significant differences in α-diversity for the Shannon index between sites with black birch as the dominant vegetation and those with red oak (figure 1E). We didn't find any significant differences for the Pielou evenness and Faith Phylogenetic Diversity tested for dominant vegetation nor for any of the other environmental variables considered (figure 1C & D). The Principal Coordinate Analysis (PCoA) using Unweighted Unifrac method shows two clusters of samples but none of the variables considered in this study explained the clustering pattern (figure 1B). PCoAs using Weighed Unifrac, Jaccard and Bray-Curtis indices don’t show any significant clustering pattern of the samples for any of the variables tested. The ANCOM-BC analysis shows significant levels of depletion and/or enrichment in relative abundance for several taxa based on vegetation and pH (p=0.05; figure 1H). Phylum *Proteobacteria* was significantly depleted in sites dominated by black birch and red oak (25.26%; p=0.05). Sites dominated by red oak and white oak showed enrichment of the phylum *Bacteroidota* (1.2%; p=0.05) and a significant depletion of the phylum *Verrucomicrobiota* (1.6%; p=0.05), in particular of *orders Pedosphaerales* (1.36%; p=0.05) and *S-BQ2-57-soil-group* (0%; p=0.05) *.* Order *Subroup_2* (12.2%; p=0.05), from class *Acidobacteriae ,* was also significantly depleted. Phylum *Bacteroidota* (1.2%) and order *Rickettsiales* (0.4%) were enriched in sites dominated by red oak and white oak *.* S-BQ2-57 soil group are uncultured bacteria in the phylum *Verrumicrobiota* not well documented. Bacteria in the order *Pedosphaerales* have been found to be important members of the microbiome in soil macroaggregates, characterized by richer labile organic matter and lower diversity (Bach et al., 2018) and in soil microbiomes of tall grass prairie (Fierer et al., 2013), but its function is not well understood. *On* sites dominated by white oak the class *Thermoleophilia,* order *Solirubrobacteriales,* from were significantly enriched (3.4%). *Solirubrobacteriales* is an order in the class *Thermoleophilia* and phylum *Actinobacteria* . Bacteria in this order are mesophylic gram positive rods commonly found is soils particularly under dry conditions and have unique adaptations to UV radiation (Jiang et al., 2023). Soil water capacity for soils where white oaks are the dominant tree is 0.2 cm/cm compared to 0.25 cm/cm for all other sites. White oaks are known to have deep root systems that makes them particularly resistant to drought conditions. We also found significant differences in relative abundance based on soil pH (p=0.05; figure 1H). Class *Thermoanaerobaculia,* phylum *Acidobacteriota,* was enriched (0.2%) and class *Acidomicrobiia,* phylum *Actinobacteriota, was* depleted (0.05%) in soils with pH 5.3 and 5.6 compared to pH 4.6 soils (figure 1H). Class *Vicinamibacteria,* phylum *Acidobacteriota,* is also significantly depleted in pH 5.6 soils (0.03%; figure 1H). Classes *Thermoanaerobaculia and Vicinamibacteria,* both members of the phylum *Acidobacteriota,* are ubiquitous in soils with reported average relative abundances of 0.92% and 3.8% respectively (McReynolds et al., 2024). Some of the members of the class *Acidomicrobiia* are extreme acidophiles with an optimal growth pH of around 2 (Hu et al., 2018).

Our results are consistent with published data supporting that soil characteristics, particularly pH, (Lauber et al., 2008; Kaiser et al., 2016) and tree species and type of leaf litter (Bonito et al., 2014; Dukunde et al., 2022; Galazka et al., 2022; Staszel-Szlachta et al., 2024; Pfeiffer et al., 2013) are determinants of microbial diversity, activity and community structure.

## Methods

**Sampling sites**

112 acres of forest located on the SUNY College at Old Westbury campus on Long Island’s North Shore. This land consists of mud, sand, gravel, and boulders eroded from Upstate New York and New England and deposited as part of the Harbor Hill Moraine and its outwash plain. Underlying bedrock is close to one hundred meters deep overlain by sediments of the Cretaceous period that were uplifted above sea level and eroded during the Tertiary period and later covered with glacial outwash and till which created the present-day landscape. The vegetation is that of a mesophytic eastern broadleaf forest that occurs on moist, well-drained sites in southeastern New York, humid temperate domain, hot continental division, and oceanic province (Bailey, 2016). The average annual temperature and precipitation for this location are 12.59 °C and 1058 mm, and length of the growing season is 165 days. The dominant trees include a mixture of five or more of the following: red oak ( *Quercus rubra* ), beech ( *Fagus grandifolia* ), black birch ( *Betula lenta* ), red maple ( *Acer rubrum* ), scarlet oak ( *Quercus coccinea* ), black oak ( *Q. velutina* ), and white oak ( *Q* . alba). There is typically a subcanopy stratum of small trees and tall shrubs dominated by flowering dogwood ( *Cornus florida* ); common associates include witchhazel ( *Hamamelis virginiana* ), sassafras ( *Sassafras albidum* ), red maple ( *Acer rubrum)* , and black cherry ( *Prunus serotina* ). We sampled twenty locations along four transects following the major axis of the forest patch in a north-south orientation.

**Soil DNA Extraction and 16S-rRNA Sequencing**

Forty topsoil samples were taken from twenty sampling sites along four transects crossing north to south the study site. All the samples were collected during the month of June in 2020 and 2021. Physico-chemical soil properties for each of the sampling sites was collected from the Web Soil Survey (https://websoilsurvey.nrcs.usda.gov/app/). Soil DNA extraction was done on the same day of collection. DNA was extracted using the Power SoilTM DNA Isolation Kit (QIAGEN Laboratories Inc., Solana Beach, USA). All 16S rRNA illumina-tag PCR reactions were performed on the DNA extracts per the Earth Microbiome Project’s protocol using 515f GTGCCAGCMGCCGCGGTAA, 806r GGACTACHVGGGTWTCTAAT primers (Walters et al. 2016; Caparoso et al., 2011 ). PCR products were pooled, and gel purified on a 2% agarose gel using the Qiagen Gel Extraction Kit (Qiagen, Germantown, Maryland, USA). Before sequencing, the purified pool was quality checked using an Agilent 2100 BioAnalyzer and Agilent DNA High Sensitivity DNA kit (Agilent Technologies, Santa Clara, California, USA). The purified pool was stored at -20˚ C and then sequenced by Wright Labs (https://www.wrightlabs.org/, Huntingdon, PA, USA) using an Illumina MiSeq v2 chemistry generating paired-end 250 base pair reads.

**16S-rRNA Sequence Bioinformatic Analysis**

Microbiome bioinformatics were performed with QIIME2-amplcion-2024.5 (Bolyen et al. 2019). Raw sequence data was imported as Casava 1.8 paired-end fastq demultiplexed files. The sequences were denoised using DADA2 (Callahan et al. 2016) (via q2‐dada2). After denoising we found 798,528 sequences corresponding to 232 different Amplicon Sequence Variants (ASV). The average number of sequences per sample was 19,963, the minimum number in a sample is 7,157 and the maximum 40,234. The mean frequency per feature was 3,442, ranging between 1000 and 39,031. All amplicon sequence variants (ASVs) were aligned with mafft (Katoh et al. 2002) (via q2‐alignment) and used to construct a phylogeny with fasttree2 (Price et al. 2010) (via q2‐phylogeny). Alpha‐diversity metrics observed features, Pielou evenness, and Faith’s Phylogenetic Diversity (Faith 1992), beta diversity metrics, weighted UniFrac (Lozupone et al. 2007), unweighted UniFrac (Lozupone et al. 2005), Jaccard distance, and Bray‐Curtis dissimilarity, and Principle Coordinate Analysis (PCoA) were estimated using q2‐diversity after samples were rarefied (subsampled without replacement) to 1000 sequences per sample. Taxonomy was assigned to ASVs using the q2‐feature‐classifier (Bokulich et al. 2018a) classify‐sklearn naïve Bayes taxonomy classifier against the Silva 138.1 habitat specific pre-trained 515f-806r-soil-non-saline classifier OTUs reference sequences (Kaehler et al., 2019, McDonald et al. 2012, Quast et al. 2013, and Robeson et al. 2013). Taxa differential abundance was done using the Analysis of Compostion of Microbobiome with Bias Correction, ANCOM-BC (Lin and Peddada, 2020).

The sequences are archived as BioProject accession number PRJNA1058532, SRX23708619-SRX23708658 in the NCBI BioProject database (https://www.ncbi.nlm.nih.gov/bioproject/?term=PRJNA1058532).

## Reagents

**Table:** 

**Name**	**Supplier**	**Catalog**
Power SoilTM DNA Isolation Kit	QIAGEN	Cat. No. 47014
QIAquick Gel Extraction Kit	Qiagen	Cat. No. 28706X4
Agilent 2100 BioAnalyzer	Agilent Technologies	NA
Agilent DNA High Sensitivity DNA	Agilent Technologies	5067-4626
Illumina MiSeq	Illumina	NA
Qiime2	https://qiime2.org/	NA
Web Soil Survey	https://websoilsurvey.nrcs.usda.gov/app/	
Forward Primer 515f	Earth Microbiome Project. Walters et al. 2016; Caparoso et al., 2011	GTGCCAGCMGCCGCGGTAA
Reverse Primer 806r	Earth Microbiome Project. Walters et al. 2016; Caparoso et al., 2011	GGACTACHVGGGTWTCTAAT

## References

[R1] Bailey Robert G. (2021). Bailey's ecoregions and subregions of the United States, Puerto Rico, and the U.S. Virgin Islands. Forest Service Research Data Archive.

[R2] Barbera Pierre, Kozlov Alexey M, Czech Lucas, Morel Benoit, Darriba Diego, Flouri Tomáš, Stamatakis Alexandros (2018). EPA-ng: Massively Parallel Evolutionary Placement of Genetic Sequences. Systematic Biology.

[R3] Bokulich Nicholas A., Kaehler Benjamin D., Rideout Jai Ram, Dillon Matthew, Bolyen Evan, Knight Rob, Huttley Gavin A., Gregory Caporaso J. (2018). Optimizing taxonomic classification of marker-gene amplicon sequences with QIIME 2’s q2-feature-classifier plugin. Microbiome.

[R4] Bolyen Evan, Rideout Jai Ram, Dillon Matthew R., Bokulich Nicholas A., Abnet Christian C., Al-Ghalith Gabriel A., Alexander Harriet, Alm Eric J., Arumugam Manimozhiyan, Asnicar Francesco, Bai Yang, Bisanz Jordan E., Bittinger Kyle, Brejnrod Asker, Brislawn Colin J., Brown C. Titus, Callahan Benjamin J., Caraballo-Rodríguez Andrés Mauricio, Chase John, Cope Emily K., Da Silva Ricardo, Diener Christian, Dorrestein Pieter C., Douglas Gavin M., Durall Daniel M., Duvallet Claire, Edwardson Christian F., Ernst Madeleine, Estaki Mehrbod, Fouquier Jennifer, Gauglitz Julia M., Gibbons Sean M., Gibson Deanna L., Gonzalez Antonio, Gorlick Kestrel, Guo Jiarong, Hillmann Benjamin, Holmes Susan, Holste Hannes, Huttenhower Curtis, Huttley Gavin A., Janssen Stefan, Jarmusch Alan K., Jiang Lingjing, Kaehler Benjamin D., Kang Kyo Bin, Keefe Christopher R., Keim Paul, Kelley Scott T., Knights Dan, Koester Irina, Kosciolek Tomasz, Kreps Jorden, Langille Morgan G. I., Lee Joslynn, Ley Ruth, Liu Yong-Xin, Loftfield Erikka, Lozupone Catherine, Maher Massoud, Marotz Clarisse, Martin Bryan D., McDonald Daniel, McIver Lauren J., Melnik Alexey V., Metcalf Jessica L., Morgan Sydney C., Morton Jamie T., Naimey Ahmad Turan, Navas-Molina Jose A., Nothias Louis Felix, Orchanian Stephanie B., Pearson Talima, Peoples Samuel L., Petras Daniel, Preuss Mary Lai, Pruesse Elmar, Rasmussen Lasse Buur, Rivers Adam, Robeson Michael S., Rosenthal Patrick, Segata Nicola, Shaffer Michael, Shiffer Arron, Sinha Rashmi, Song Se Jin, Spear John R., Swafford Austin D., Thompson Luke R., Torres Pedro J., Trinh Pauline, Tripathi Anupriya, Turnbaugh Peter J., Ul-Hasan Sabah, van der Hooft Justin J. J., Vargas Fernando, Vázquez-Baeza Yoshiki, Vogtmann Emily, von Hippel Max, Walters William, Wan Yunhu, Wang Mingxun, Warren Jonathan, Weber Kyle C., Williamson Charles H. D., Willis Amy D., Xu Zhenjiang Zech, Zaneveld Jesse R., Zhang Yilong, Zhu Qiyun, Knight Rob, Caporaso J. Gregory (2019). Reproducible, interactive, scalable and extensible microbiome data science using QIIME 2. Nature Biotechnology.

[R5] Bonito Gregory, Reynolds Hannah, Robeson Michael S., Nelson Jessica, Hodkinson Brendan P., Tuskan Gerald, Schadt Christopher W., Vilgalys Rytas (2014). Plant host and soil origin influence fungal and bacterial assemblages in the roots of woody plants. Molecular Ecology.

[R6] Bray J. Roger, Curtis J. T. (1957). An Ordination of the Upland Forest Communities of Southern Wisconsin. Ecological Monographs.

[R7] Callahan Benjamin J, McMurdie Paul J, Rosen Michael J, Han Andrew W, Johnson Amy Jo A, Holmes Susan P (2016). DADA2: High-resolution sample inference from Illumina amplicon data. Nature Methods.

[R8] Caporaso JG, Lauber CL, Walters WA, Berg-Lyons D, Lozupone CA, Turnbaugh PJ, Fierer N, Knight R. 2010. Global patterns of 16S rRNA diversity at a depth of millions of sequences per sample. Proceedings of the National Academy of Sciences 108: 4516-4522. 10.1073/pnas.1000080107 PMC3063599 20534432

[R9] Cardenas Erick, Kranabetter J M, Hope Graeme, Maas Kendra R, Hallam Steven, Mohn William W (2015). Forest harvesting reduces the soil metagenomic potential for biomass decomposition. The ISME Journal.

[R10] Czech Lucas, Barbera Pierre, Stamatakis Alexandros (2020). Genesis and Gappa: processing, analyzing and visualizing phylogenetic (placement) data. Bioinformatics.

[R11] Donoso, J. A. P. Perspectives on temperate forest management. *Proceedings of the XI World Forestry Congress* **38,** 153-162 (1997).

[R12] Dukunde Amélie, Schneider Dominik, Schmidt Marcus, Veldkamp Edzo, Daniel Rolf (2019). Tree Species Shape Soil Bacterial Community Structure and Function in Temperate Deciduous Forests. Frontiers in Microbiology.

[R13] Faith Daniel P. (1992). Conservation evaluation and phylogenetic diversity. Biological Conservation.

[R14] Forseth, I. (2010) Terrestrial Biomes. Nature Education Knowledge 3(10):11

[R15] Gałązka Anna, Marzec-Grządziel Anna, Varsadiya Milan, Niedźwiecki Jacek, Gawryjołek Karolina, Furtak Karolina, Przybyś Marcin, Grządziel Jarosław (2022). Biodiversity and Metabolic Potential of Bacteria in Bulk Soil from the Peri-Root Zone of Black Alder (Alnus glutinosa), Silver Birch (Betula pendula) and Scots Pine (Pinus sylvestris). International Journal of Molecular Sciences.

[R16] Hu Danyu, Cha Guihong, Gao Beile (2018). A Phylogenomic and Molecular Markers Based Analysis of the Class Acidimicrobiia. Frontiers in Microbiology.

[R17] Kaehler Benjamin D., Bokulich Nicholas A., McDonald Daniel, Knight Rob, Caporaso J. Gregory, Huttley Gavin A. (2019). Species abundance information improves sequence taxonomy classification accuracy. Nature Communications.

[R18] Kaiser Kristin, Wemheuer Bernd, Korolkow Vera, Wemheuer Franziska, Nacke Heiko, Schöning Ingo, Schrumpf Marion, Daniel Rolf (2016). Driving forces of soil bacterial community structure, diversity, and function in temperate grasslands and forests. Scientific Reports.

[R19] Katoh K. (2002). MAFFT: a novel method for rapid multiple sequence alignment based on fast Fourier transform. Nucleic Acids Research.

[R20] Keenan Rodney J., Reams Gregory A., Achard Frédéric, de Freitas Joberto V., Grainger Alan, Lindquist Erik (2015). Dynamics of global forest area: Results from the FAO Global Forest Resources Assessment 2015. Forest Ecology and Management.

[R21] Lauber Christian L., Strickland Michael S., Bradford Mark A., Fierer Noah (2008). The influence of soil properties on the structure of bacterial and fungal communities across land-use types. Soil Biology and Biochemistry.

[R22] Lin Huang, Peddada Shyamal Das (2020). Analysis of compositions of microbiomes with bias correction. Nature Communications.

[R23] Lladó Salvador, López-Mondéjar Rubén, Baldrian Petr (2018). Drivers of microbial community structure in forest soils. Applied Microbiology and Biotechnology.

[R24] Lladó Salvador, López-Mondéjar Rubén, Baldrian Petr (2017). Forest Soil Bacteria: Diversity, Involvement in Ecosystem Processes, and Response to Global Change. Microbiology and Molecular Biology Reviews.

[R25] Lozupone Catherine A., Hamady Micah, Kelley Scott T., Knight Rob (2007). Quantitative and Qualitative β Diversity Measures Lead to Different Insights into Factors That Structure Microbial Communities. Applied and Environmental Microbiology.

[R26] Lozupone Catherine, Knight Rob (2005). UniFrac: a New Phylogenetic Method for Comparing Microbial Communities. Applied and Environmental Microbiology.

[R27] Martinović Tijana, Odriozola Iñaki, Mašínová Tereza, Doreen Bahnmann Barbara, Kohout Petr, Sedlák Petr, Merunková Kristina, Větrovský Tomáš, Tomšovský Michal, Ovaskainen Otso, Baldrian Petr (2021). Temporal turnover of the soil microbiome composition is guild‐specific. Ecology Letters.

[R28] McDonald Daniel, Price Morgan N, Goodrich Julia, Nawrocki Eric P, DeSantis Todd Z, Probst Alexander, Andersen Gary L, Knight Rob, Hugenholtz Philip (2011). An improved Greengenes taxonomy with explicit ranks for ecological and evolutionary analyses of bacteria and archaea. The ISME Journal.

[R29] McReynolds Ella, Elshahed Mostafa S., Youssef Noha H. (2024). An ecological-evolutionary perspective on the genomic diversity and habitat preferences of the Acidobacteriota.

[R30] MIRARAB S., NGUYEN N., WARNOW T. (2011). SEPP: SATé-Enabled Phylogenetic Placement. Biocomputing 2012.

[R31] Nemergut Diana R., Schmidt Steven K., Fukami Tadashi, O'Neill Sean P., Bilinski Teresa M., Stanish Lee F., Knelman Joseph E., Darcy John L., Lynch Ryan C., Wickey Phillip, Ferrenberg Scott (2013). Patterns and Processes of Microbial Community Assembly. Microbiology and Molecular Biology Reviews.

[R32] Parks Donovan H., Tyson Gene W., Hugenholtz Philip, Beiko Robert G. (2014). STAMP: statistical analysis of taxonomic and functional profiles. Bioinformatics.

[R33] Peng Ziheng, Liang Chunling, Gao Min, Qiu Yu, Pan Yanjing, Gao Hang, Liu Yu, Li Xiaomeng, Wei Gehong, Jiao Shuo (2022). The neglected role of micronutrients in predicting soil microbial structure. npj Biofilms and Microbiomes.

[R34] Pfeiffer Birgit, Fender Ann-Catrin, Lasota Sandra, Hertel Dietrich, Jungkunst Hermann F., Daniel Rolf (2013). Leaf litter is the main driver for changes in bacterial community structures in the rhizosphere of ash and beech. Applied Soil Ecology.

[R35] Prada-Salcedo Luis Daniel, Prada-Salcedo Juan Pablo, Heintz-Buschart Anna, Buscot François, Goldmann Kezia (2022). Effects of Tree Composition and Soil Depth on Structure and Functionality of Belowground Microbial Communities in Temperate European Forests. Frontiers in Microbiology.

[R36] Price Morgan N., Dehal Paramvir S., Arkin Adam P. (2010). FastTree 2 – Approximately Maximum-Likelihood Trees for Large Alignments. PLoS ONE.

[R37] Quast Christian, Pruesse Elmar, Yilmaz Pelin, Gerken Jan, Schweer Timmy, Yarza Pablo, Peplies Jörg, Glöckner Frank Oliver (2012). The SILVA ribosomal RNA gene database project: improved data processing and web-based tools. Nucleic Acids Research.

[R38] Robeson Michael S., O’Rourke Devon R., Kaehler Benjamin D., Ziemski Michal, Dillon Matthew R., Foster Jeffrey T., Bokulich Nicholas A. (2021). RESCRIPt: Reproducible sequence taxonomy reference database management. PLOS Computational Biology.

[R39] Staszel-Szlachta Karolina, Lasota Jarosław, Szlachta Andrzej, Błońska Ewa (2024). The impact of root systems and their exudates in different tree species on soil properties and microorganisms in a temperate forest ecosystem. BMC Plant Biology.

[R40] Suzuki Yohey, Kelly Shelly D., Kemner Ken M., Banfield Jillian F. (2004). Enzymatic U(VI) reduction by
*Desulfosporosinus*
species. Radiochimica Acta.

[R41] Thoms Carolin, Gattinger Andreas, Jacob Mascha, Thomas Frank M., Gleixner Gerd (2010). Direct and indirect effects of tree diversity drive soil microbial diversity in temperate deciduous forest. Soil Biology and Biochemistry.

[R42] Uroz S., Buée M., Deveau A., Mieszkin S., Martin F. (2016). Ecology of the forest microbiome: Highlights of temperate and boreal&nbsp;ecosystems. Soil Biology and Biochemistry.

[R43] Vasseur, L. (2012) Restoration of Deciduous Forests. *Nature Education Knowledge* 3(12):1

[R44] Walters William, Hyde Embriette R., Berg-Lyons Donna, Ackermann Gail, Humphrey Greg, Parada Alma, Gilbert Jack A., Jansson Janet K., Caporaso J. Gregory, Fuhrman Jed A., Apprill Amy, Knight Rob (2016). Improved Bacterial 16S rRNA Gene (V4 and V4-5) and Fungal Internal Transcribed Spacer Marker Gene Primers for Microbial Community Surveys. mSystems.

[R45] Wood Stephen A., Gilbert Jack A., Leff Jonathan W., Fierer Noah, D'Angelo Heather, Bateman Carling, Gedallovich Seren M., Gillikin Caitlyn M., Gradoville Mary R., Mansor Patahayah, Massmann Audrey, Yang Nina, Turner Benjamin L., Brearley Francis Q., McGuire Krista L. (2017). Consequences of tropical forest conversion to oil palm on soil bacterial community and network structure. Soil Biology and Biochemistry.

